# Identification of an early diagnostic biomarker of lung adenocarcinoma based on co-expression similarity and construction of a diagnostic model

**DOI:** 10.1186/s12967-018-1577-5

**Published:** 2018-07-20

**Authors:** Zhirui Fan, Wenhua Xue, Lifeng Li, Chaoqi Zhang, Jingli Lu, Yunkai Zhai, Zhenhe Suo, Jie Zhao

**Affiliations:** 1 Department of Pharmacy, The First Affiliated Hospital of Zhengzhou University, Zhengzhou, 450052 Henan China; 2 Cancer Center, The First Affiliated Hospital of Zhengzhou University, Zhengzhou, 450052 Henan China; 3 Center of Telemedicine, The First Affiliated Hospital of Zhengzhou University, Zhengzhou, 450052 Henan China; 4 Engineering Laboratory for Digital Telemedicine Service, Zhengzhou, 450052 Henan China

**Keywords:** Lung adenocarcinoma, Early diagnostic, Diagnostic model

## Abstract

**Background:**

The purpose of this study was to achieve early and accurate diagnosis of lung cancer and long-term monitoring of the therapeutic response.

**Methods:**

We downloaded GSE20189 from GEO database as analysis data. We also downloaded human lung adenocarcinoma RNA-seq transcriptome expression data from the TCGA database as validation data. Finally, the expression of all of the genes underwent z test normalization. We used ANOVA to identify differentially expressed genes specific to each stage, as well as the intersection between them. Two methods, correlation analysis and co-expression network analysis, were used to compare the expression patterns and topological properties of each stage. Using the functional quantification algorithm, we evaluated the functional level of each significantly enriched biological function under different stages. A machine-learning algorithm was used to screen out significant functions as features and to establish an early diagnosis model. Finally, survival analysis was used to verify the correlation between the outcome and the biomarkers that we found.

**Results:**

We screened 12 significant biomarkers that could distinguish lung cancer patients with diverse risks. Patients carrying variations in these 12 genes also presented a poor outcome in terms of survival status compared with patients without variations.

**Conclusions:**

We propose a new molecular-based noninvasive detection method. According to the expression of the stage-specific gene set in the peripheral blood of patients with lung cancer, the difference in the functional level is quantified to realize the early diagnosis and prediction of lung cancer.

## Background

Among patients with non-small cell lung cancer(NSCLC), which accounts for approximately 85% of all lung cancer cases, nearly 50% have lung adenocarcinoma, which is the most common lung cancer [1]. Lung adenocarcinoma usually begins at the outer part of the lung tissue. Early lung cancer does not yield significant clinical manifestations; only in the late stage will there be a gradual emergence of chronic coughs, bloody sputum, and other symptoms. In that case, some early symptoms (such as fatigue, shortness of breath, or upper back and chest pain) are likely to be overlooked [2].

The clinical diagnosis of lung cancer is mainly dependent on an X-ray; lung cancer lesions often appear as abnormal shadows on X-rays. However, in up to 25% of lung cancer cases, the chest X-ray does not reveal any abnormal lesions and returns a perfect “normal” diagnosis. If you still suspect cancer, you can also use other more sensitive diagnostic methods, including computed tomography (CT) or MRI scans [3, 4]. According to the results, the doctor may wish to obtain a lung tissue sample by using a puncture to confirm. However, there are many controversies associated with this invasive detection method. First, nobody can guarantee that puncture sampling will always obtain tumour cells, and the invasion of the sampling process has a potential risk of cancer metastasis. Therefore, a new blood detection method called liquid biopsy has emerged, and it can accurately detect the expression of specific genes in lung adenocarcinoma under noninvasive conditions. According to the appearance of particular genes in the peripheral blood of patients, this new detection method will achieve the early diagnosis of lung cancer with 91% accuracy and long-term monitoring of therapeutic response [5]. Beyond that, this gene detection method has a high sensitivity and specificity compared with traditional detection methods while avoiding the risk of cancer cell proliferation due to invasive exposure.

Once the doctor clinically confirms that a patient has lung cancer, the doctor will classify the result into different stages based on the progress of lung cancer, such as whether it has spread, and if any other articles may be involved [6]. Multi-staging helps the direct treatment in a more appropriate manner, neither under treating a malignancy or over treating it and causing more harm than good. According to the malignant degree of carcinoma, there will be four stages: stage I, stage II, stage III and stage IV [7]. Stage I: the cancer is localized and has not spread to any lymph nodes. Stage 2: the cancer has spread to the lymph nodes, the lining of the lungs, or the significant passageways of the lungs. Stage 3: the cancer has spread to nearby tissue. Stage 4: the cancer has spread (metastasized) to farther reaches of the body. Lung adenocarcinoma is classified by the qualitative analysis of cancer tissue after diagnosis, which means that in the diagnosis stage, there is no distinction between the stages of lung adenocarcinoma [8].

This process also leads to many lung cancer patients not knowing whether they are probably in an advanced stage. By combining the genetic analyses at the cellular and functional level, experimenters identify specific genes and mutations in the four stages. These genes may be the markers of different stages of lung cancer or the target for personalized treatment. Additionally, the variability function explains the biological difference between the various stages and the malignant mechanism of the fatal development of cancer to a certain extent.

In conclusion, the traditional method of finding disease markers is mostly dependent on the expression level of single genes. The assumption is that each gene is relatively independent, so the doctor will use the selected gene’s characteristics to design diagnostic kits, make the combination of chips or sequencing, and predict the risk of illness based on gene expression [9]. However, in organisms, the gene is not relatively independent, and there is a functional interaction. By considering the patient’s molecular-level and functional-level changes from a higher perspective, this study converts gene expression information into a functional imbalance. On the one hand, this method overcomes the instability of cross-sample single gene markers. On the other hand, it prompts the underlying pathogenesis from the functional level, while the gene with the specific function we collected is likely to be a clinically significant therapeutic target or diagnostic marker. More importantly, our analysis found 12 genes that not only can predict lung cancer patients in the early phase but can also present dynamic changes during cancer development. Moreover, patients carrying variations in these genes tend to have a poor outcome in terms of survival status compared with patients without variations.

## Methods

### Data remodelling and grouping

The RNA-seq data GSE20189 were downloaded from the GEO database [10]. We first split the lung cancer data into groups based on the clinical data. The original data consistes of 22,277 genes and 162 samples. There were 81 controls, 28 patients in the early phase (stage I) and 53 patients in the late phase (stage II–stage IV).

### Data normalization

Initially, we accomplish the data pre-processing and standardization, and we removed genes and samples with the ratio of missing values higher than 10%. The missing values within remaining samples are replaced with the mean values of the corresponding genes in the other samples. We calculated the mean value and standard deviation of each gene in the control group. Then, we applied Z-score normalization [11] to all samples after which the expression of the gene in the control group obeys the standard normal distribution, with a mean value of 0 and a variance of 1. Therefore, for gene I, if there is no difference between different stages of the lung adenocarcinoma samples, the case groups should be subject to a normal distribution. Otherwise, we believe the gene i in a specific stage is obviously different compared to the control group; this differentially expressed gene is likely to act as a crucial biomarker in early diagnosis.

### Stage grading-specific gene extraction

In the evolutionary process, most of the genes are conservative and commonly do not have an apparent difference in expression under the disease signal stimulation. If the gene is significantly associated with lung adenocarcinoma, then different expression should be observed in at least one stage group. Therefore, we used the coefficient of variation to assess the fluctuation of genes across the lung adenocarcinoma samples. The coefficient of variation can be calculated by Eq. 1.

1 $$CV = \frac{sd}{mean}$$

Mean is the mean expression of the gene in all lung adenocarcinoma samples, and sd is the corresponding standard deviation. According to the distribution of all the coefficients of variation in the genes, we only screened genes whose absolute value of CV rank in the top 50% as genes that may be associated with lung adenocarcinoma. The remaining 50% of the genes are due to a small fluctuation in the vicinity of 0; therefore, these genes are supposed to have little impact on lung adenocarcinoma. To identify the specific genes for early or late phase, we used the limma method [12] to evaluate the differences compared with control group. The significance threshold of the P value was set to 0.05, and the cut off of the absolute value of the fold change was set to 1. The significantly differentially expressed genes in the early phase were marked as Δ0. The differentially expressed genes related to the late phase were marked as Δ1. The intersection between early and late phase specific genes was marked as Δ2. These genes can distinguish normal and cancer patients in the early phase and present dynamic changes with cancer development.

### Co-expression correlation analysis

Since the interaction between genes changes from one stage to the next stage of lung adenocarcinoma, the two correlated genes tend to have a common biological effect in the non-disease state from the biological point of view. It appears as a mutual synergy or interaction. However, in the disease state, the function of the genes is abnormal, which may results in the change of correlation. Therefore, we examined the expression correlation in Δ0, Δ1 and Δ2 using the Pearson correlation coefficient [13] (greater than 0.5 is positively correlated, and below − 0.5 is negatively correlated).

### Unsupervised clustering analysis

We utilize correlation analysis to construct the correlation coefficient matrix between genes and take advantage of hierarchical clustering [14] to achieve unsupervised clustering for samples and genes. From the results of unsupervised clustering, we observe and analyse the effect of distinguishing cancer patients from controls at the gene level. On the one hand, we can verify the Δ2 gene’s ability to identify the specific stage of the lung adenocarcinoma sample; on the other hand, we can observe the gene expression patterns at different stages, such as genes in stage i that have a high expression in a group while having a low expression in stage j. The transformation of this expression pattern also indicates that the progression of the disease affects the function of gene regulation, which leads to some function level changes. The clustering results are visualized using heatmap thermal maps [15].

### Specific and non-specific co-expression network analysis

Because the correlation between gene expression is different through disease status, the status specific networks should also reflect a significant difference in network characteristics from the perspective of the system network. We constructed specific networks of control, early and late phase based on the co-expression relationship between genes from Δ0 and Δ1. The non-specific network is constructed using genes from Δ2. If there are co-expression relationships between the two genes, there is an edge between genes. Because of the co-expression network constructed by gene co-expression in different disease states, it shows a significantly different topological nature, suggesting that the signal transmission efficiency of the system network is remarkably unlike in different vicious grades. Hence, we start analysing from six topological properties of network connectivity [16], namely, ASLP (average shortest path), Closeness Centrality, Cluster Coefficient, and Degree Distribution. If the edge of the network is missing, that is, the co-expression relationship between the genes disappears, the average shortest path of the network increases, and along with a decrease in the Degree Distribution, Clustering Coefficient, and Closeness Centrality, the network signal transmission performance is declining as well. The specific network of the control status reflects intrinsic relationships among genes. The specific network of the early phase reflects the changes in the initial stage of cancer. The specific network of the late phase reflects more variations occurring among genes. On the other hand, as the overlapped genes (Δ2) present deviations in both early and late phase, these genes can be regard as biomarkers associated with cancer progression. Therefore, we also construct a non-specific network using Δ2. Eventually, we use the degree of distribution of gene nodes in the network to evaluate the importance of genes; the higher the degree of the gene, the more adjacent genes are becoming affected by the abnormal gene. We convert the degree of all genes through the sigmoid function [17] to the weight of 0–1, and the weight of the gene not in the network defaults to the minimum.

2 $${\text{sigmoid}}\;\left( {\text{degree}} \right) = \frac{1}{{1 + e^{ - degree} }}$$

### Functional pathway enrichment

To further analyse the biological functions involved in the specific genes in the different disease states from the functional level, we apply the functional enrichment analysis on the Δ2 genes. By using Fisher’s exact test [18], the significant pathways selected are the functions for regulation of these specific genes. Since these genes have dissimilar expression patterns in the different stages, at the same time, the genes that have a co-expression correlation tend to participate in the same biological ability; we deduce that these pathways can better indicate the efficacy of the genes in distinguishing stages. Beyond that, these pathways with abnormal functional levels in different stages can be used to explain the mechanisms of disease progression, and these pathways may contain potential drug targets or diagnostic markers.

### Identification of significantly deviated pathways

Suppose we used the intersection gene to obtain N pathways through function enrichment. We first identify the differentially expressed genes in each gene pool and then use the inverse cumulative distribution function to convert the P value of ANOVA [19] to the Z value and multiply the weight of the gene. The differentially expressed genes in pathway P are calculated from the formula to calculate the deviation score A (P) of the pathway [20], as follows:

3 $$\begin{aligned} A(p_{j} ) = \hbox{max} \left\{ {\frac{1}{\sqrt t }\sum\limits_{i = 1}^{t} {Z^{\prime}_{{j_{i} }} \left| {1 \le t \le k} \right.} } \right\} \hfill \\ A_{\text{corrected}} (p_{j} ) = \frac{{A(p_{j} ) - \mu_{k} }}{{\sigma_{k} }} \hfill \\ \end{aligned}$$

In the calculation process, we first sort the differentially expressed genes from large to small; therefore, the larger the Z value is, the more significant is the gene expressed. Suppose that there are k differentially expressed genes in pathway P, then 2, 3……k genes are used to calculate the Z score mean value. When the Z score of the t genes is the maximum, the corresponding t genes have the greatest contribution to pathway P, and we calculate the deviation score A(P) of pathway P in the disease state.

To eliminate the influence of the size of the pathway itself, we calibrate the A(P) score with the correction method of the random perturbation principle. For the pathway P, the deviation score is A(P), and we recalculate a new A(p)’ by k sets of genes in the random selected pathways. After 10,000 random cycles, we calculate the mean μ and standard deviation of the A(p)’ background distribution. A calibrated A_corrected_ is obtained using Eq. 3.

There are some problems to be considered when calculating the deviation score of pathway P. First, the number of genes differentially expressed in the pathway is large, but not all of them have a significant impact on the pathway, such as some genes belonging downstream of this pathway. The difference in expression of these genes is likely due to changes in upstream abnormal signals. In contrast, certain upstream genes, important enzymes, transporters, and other genes that have important regulatory effects may have a greater impact on the pathway. Second, the number of the genes in each pathway is significantly different; in order to eliminate the impact of the size of a certain pathway itself, we utilize the random punctuation process.

### Early screening gene filtering using RFE

Significant pathways identified from random permutation present functional differences in the early and late phases of lung cancer development. This further suggests that the genes regulating these pathways have important roles in lung cancer. On the one hand, lung adenocarcinoma-related genes exhibit differences in the level of expression under the disease signal stimulation. On the other hand, the co-expression relation changes between the genes and thus affects the levels of downstream functional pathways. These genes involved in the regulation may be potential targets for lung cancer treatment or new clinical monitoring and diagnostic indicators. To accurately identify the optimal combination of gene features, we used the recursive feature elimination (RFE) algorithm [21] to filter genes. Finally, we screened out the risk-related genes of lung cancer to train the diagnostic model.

### Establishing an early diagnostic model

Actually, as the SVM performs better in the binary classification, we combined stage I and stage II as the early benign group, while stage III and stage IV as the advanced malignant group. To distinguish patients belonging to benign and malignant groups using risk-related genes as features, we utilized the supervised classifier support vector machine (SVM) to train a diagnostic model [22]. Default parameters were used to initialize the model, including the RBF nonlinear kernel function, gamma of 0 and so on. A grid search algorithm was used to optimize all parameters [23]. Cross validation was used to calculate true positive rates and false positive rates, and an ROC curve was drawn to estimate the model’s performance.

### Survival analysis of independent validation data

To further validate the early risk genes, our screen can not only achieve the early diagnosis of lung cancer but also estimate the prognosis of patients to a certain extent, to provide the basis for the individual treatment strategy. We downloaded the lung adenocarcinoma samples from the TCGA database as independent validation data. We used a Cox regression analysis of the lung adenocarcinoma sample’s overall survival data based on the risk genes. The P value was calculated to estimate the correlation between the survival time and risk gene variance.

## Results

### Phase-specific gene recognition

**Fig. 1:**
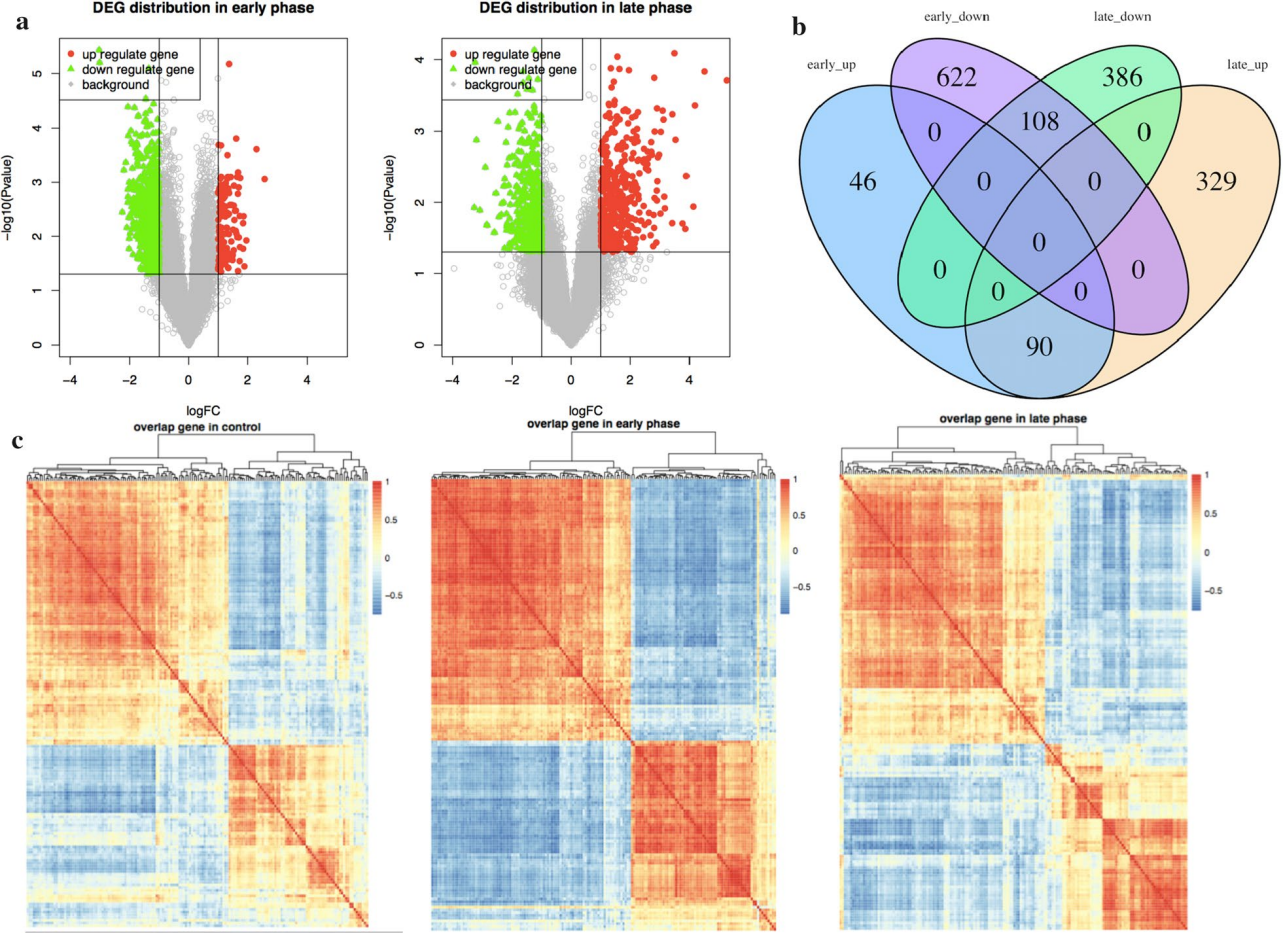
The distribution of differentially expressed genes in the early and late phase. **a** The horizontal axis is logFC, and the vertical axis is the P-value after the negative logarithm conversion. The left panel shows the distribution of early-phase lung cancer related genes, and the right panel shows the distribution of late-phase lung cancer related genes. Up- and down-regulated genes are marked in red and green, respectively. **b** Up/down-regulated genes in the early and late phases are marked with four colour markers. **c** Each colour block corresponds to the correlation coefficient of two genes: the red represents a positive correlation, and the blue represents a negative correlation. The heatmap matrix from left to right corresponds to the control and the early and late phases

We used lung adenocarcinoma samples of the early phase and late phase late to compare with healthy control groups, respectively. According to the result of the limma algorithm, 866 early phase-related differentially expressed genes were identified, including 136 up-regulated genes and 730 down-regulated genes; 913 late phase-related differentially expressed genes were identified, of which 419 were down-regulated genes and 494 were up-regulated genes. The distribution of the two groups of differentially expressed genes at the P value and logFC levels is shown in Fig. 1a.

In Fig. 1a, the down-regulated genes are labelled with green, and the up-regulated genes are labelled with red. It can be observed from the figure that the down-regulated genes are dominant in the early stage, whereas the up-regulated genes are dominant in the late phase as the cancer progresses. This suggests that an increasing number of genes are up-regulated during the malignant process of lung adenocarcinoma. The intersection genes of the early phase and late phase are shown in Fig. 1b.

We found 108 shared down-regulated genes in early and late lung cancer-related genes as well as 90 shared up-regulated genes. This indicates that the expression levels of these 198 genes in early and late lung cancer patients are both different. In the process of the malignant progression of lung adenocarcinoma, the 198 genes showed a dynamic change. On the one hand, this dynamic pattern of genes associated with the disease can be used as a clinical indicator to achieve an early diagnosis. On the other hand, the function of these genes is also likely to regulate the progress of cancer.

### Co-expression correlation analysis

If the intrinsic correlation of a pair of genes is absent after entering a disease stage, this indicates one of these genes is associated with lung cancer. In contrast, if a pair of non-related genes, appears to be correlated in disease status, then it suggests that this two genes are likely initially divided into two parallel pathways. In the process of lung cancer progression, one of the pathways is dysfunctional; therefore, another pathway is activated as a compensatory pathway, thus reflecting the new gene co-expression relationship [24]. Other than these two groups of co-expression patterns, the remaining groups in any stage in the stable co-expression are those genes that always maintain the relationship along with disease progression. Therefore, it is essential to identify the co-expressed genes of these three different patterns, which is useful for explaining the specificity of the lung adenocarcinoma stage. In cancer studies, co-expression correlations between genes are more important because the correlation between genes varies dynamically with cancer progression. This dynamic change, on the one hand, provides a basis for the pathogenesis of cancer progression and, on the other hand, is also an important feature of dynamic monitoring of patient status. We use the Pearson algorithm to internally compute the correlation coefficient between any two genes for each stage-specific gene set.

Table 1 calculates the number of genes in the phase-specific gene sets that have a remarkable correlation. Most of the relevant genes are positively correlated, and a few genes are negatively correlated. At the same time, we found that there are 3015 gene pairs in the three phases’ intersections across the normal and cancer status. These gene pairs are stable in the expression of relevance within the three phases; 3015 gene pairs involve 164 genes in total, and therefore, we investigate the 164 genes in the three phases of the co-expression state, as shown in Fig. 1c.

**Table 1:** Correlated gene pair numbers in three phases

	Positive	Negative	Total
Control	3023	504	3527
Early phase	5267	4431	9698
Late phase	3767	889	4659
Overlap	3015	0	3015

The first column lists three phases and intersections; columns 2–4 correspond to the positive correlation, the negative correlation, and the related genes in total

We observe that the 164 genes of the intersection reflect apparent differences in the four stages. Most genes are still positively correlated, but the degree and type of correlation between any two genes are not consistent in the different stages. This result again suggests that the covariant correlation between the two genes changes with the progression of lung adenocarcinoma.

### Unsupervised clustering analysis

**Fig. 2:**
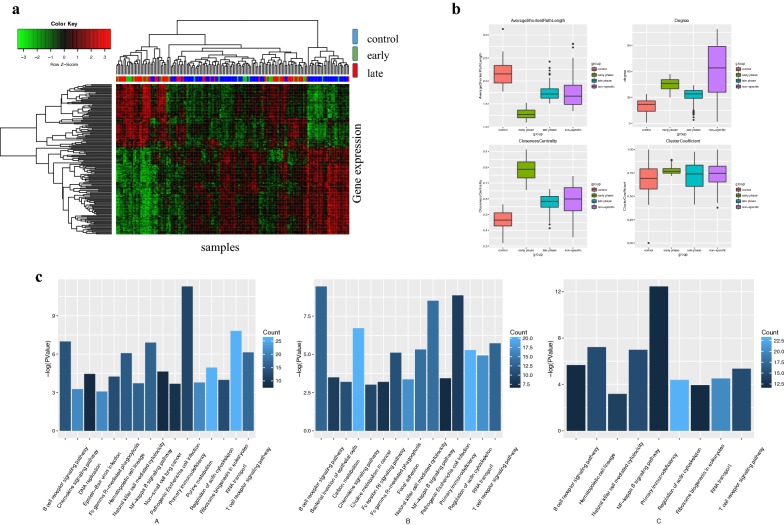
The expression and function property of the genes. **a** The horizontal axis represents the sample; the vertical axis represents the genes. We use three colours to mark the different levels of classification of lung adenocarcinoma: blue for the control group, green for the early phase, and red for the late phase. The red and green blocks represent the expression pattern of the gene; green represents low expression, and red represents high expression. **b** shows the analysis of four topological network properties, including the average shortest path, node degree distribution, closeness centrality, and clustering coefficient. Different groups are marked in different colours. **c** A–C corresponds to the pathway enrichment results of three phases. The horizontal axis is the pathway term, and the vertical axis is the P-value after the negative logarithmic transformation. We label the number of genes in the pathway by dark blue and light blue markers. The brighter the colour, the more genes that are enriched in the pathway; the darker the colour, the fewer genes that are enriched

Since the 164 specific genes shared by the early and late phases are expressed differently during the cancer process, it is an important feature to monitor the progression of the disease. Therefore, we take advantage of the 164 genes of the intersection to perform a cluster analysis on the three groups. By using the Pearson correlation coefficient, we construct a correlation matrix, and for the clustering method, we use hierarchical clustering. Unsupervised clustering analysis is performed on all the samples to examine the discriminant effects of these genes on different phase samples. The results are shown in Fig. 2a.

We can intuitively observe that almost all of the cancer samples are clustered together, and the control samples are clustered together. Therefore, we can conclude that at the 164 genes’ molecular level, the control and cancer samples have remarkable differences. However, early- and late-phase cancer samples are mixed together and are not easy to distinguish. It is also found that the expression pattern of the gene changes significantly from the normal state to the tumour state. The genes that are highly expressed in the control group have a low expression level in lung cancer samples, and vice versa. At the same time, we also found that a certain proportion of lung adenocarcinoma samples and other cancer samples are not clustered together but are mixed in the control sample. This suggests that some samples, although defined as being from lung cancer patients, have a molecular level that is close to the healthy control sample and that these samples are likely to have a better prognosis.

### Specific and non-specific co-expression of network analysis

We continue to construct specific and non-specific networks in different phases. In networks, the gene is a node, and the correlation is an edge; if the two genes are positively correlated, then the edge is red, and if the correlation is negative, the edge becomes green. The Cytoscape software realizes the network construction, and the network topology is analysed by using the network analysis plug-in.

The edge between nodes represents the correlation coefficient; the stronger the correlation is, the thicker the edge. It is clear that in each phase-specific network, some genes assemble into clusters. There is a significant co-expression correlation between the genes within each cluster, indicating that the genes within the cluster may be functionally consistent. We use the network analysis plug-into do the topology analysis for the four networks separately, and the results are shown in Fig. 2b.

The average shortest path measures the average state of the shortest path of a node to the other nodes in the network. Therefore, the shorter the average shortest path is, the more convergent the network and the higher the signal transmission efficiency. The degree distribution is a measure of the number of adjacent nodes in the network. The higher the degree, the higher the number of adjacent nodes that can affect the gene and the higher the signal transmission efficiency. Closeness centrality reflects the proximity of a node to other nodes in the network; the closer to the centre, the stronger the network shrinkage is and the closer the distance is between genes. The clustering coefficient is a sub-module that represents the ability of adjacent nodes to form a complete graph in a graph, a high clustering coefficient, and a sub-module that may exist in the network.

**Fig. 3:**
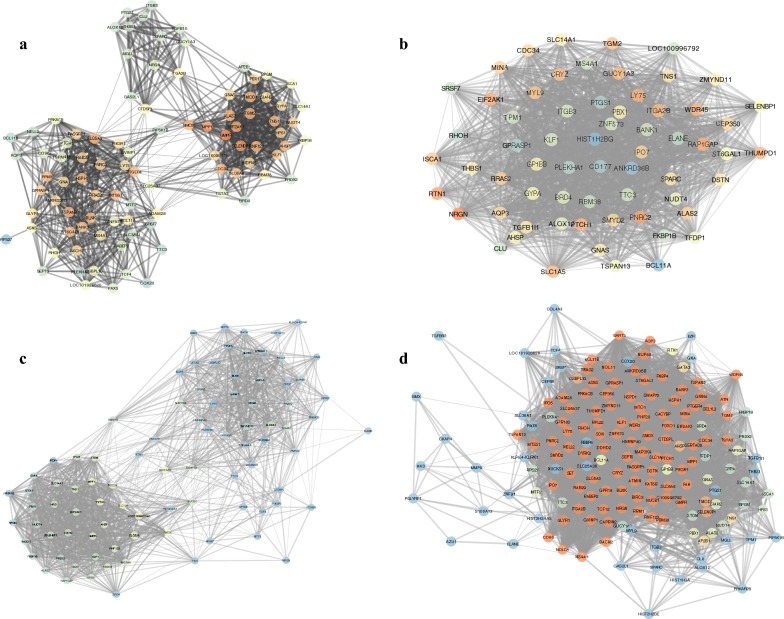
The specific and nonspecific coexpression networks. The specific and non-specific networks of different phases, from **a** to **d**, corresponding to control, early-, and late-phase related genes and the overlapping gene set. The closer the node colour is to the blue, the higher degree the node has in the network; the closer the node colour is to red, and the lower degree the node has

It can be seen from Fig. 2b that the changes in the early phase of lung adenocarcinoma in three specific networks are the most obvious, which is reflected in the average shortest path, the increase in the degree, the clustering coefficient and the closeness centrality. This series of network topological features suggests that in patients with early lung cancer signal stimulation, the patient’s body produces a significant stress response to resist and compensate for abnormal molecular function. In the early stage of lung cancer, the specific network is obviously contracted, thus reducing the average shortest path, increasing the clustering coefficient and approaching the centre, and improving the performance of the network signal transmission as a whole. However, with the further progress of lung cancer, this stress response in the early phase disappears or is insufficient to compensate for functional abnormalities and reflected in a gradually decreased network performance. The average performance of a non-specific network is between the normal and disease states, and because of the greater number of nodes and edges in nonspecific networks, the degree distributions are relatively more discrete. Detailed network structures and genes information of specific or non-specific networks can be seen from Fig 3.

### Functional pathway enrichment analysis

We apply a functional enrichment analysis to each stage-specific gene set. For the analytical method, we adopt the Fisher method, and the significance threshold is P < 0.05. We obtain the significance (P) of the path involved in each stage and the number of genes enriched in the corresponding pathway. The results are shown in Fig. 2c.

The three graphs in Fig. 2c correspond to early phase genes, late phase genes and their intersections. By observation, the significant functions involved in the early phase include primary immunodeficiency and RNA transport. The specific functions of the late phase concentrate in the T/B cell receptor signalling pathway, chemokine signalling pathway, NF-kappa B signalling pathway and primary immunodeficiency. Functions related to intersection genes concentrate on primary immunodeficiency. Functional enrichment analysis suggests that the mechanism of immune regulation in the process of lung adenocarcinoma significantly changes. Additionally, abnormal immune systems include innate immunity, T/B lymphocyte-regulated specific adaptive immunity, natural killer-regulated nonspecific immunity, and other infections and inflammation-related functions. This further suggests that abnormalities of the immune system are essential causes of the progression of lung adenocarcinoma.

Moreover, combined with the network topology in the early and late phase changes in lung cancer, we speculated that during the early phase of lung cancer, the collective immune system in the stress response process plays an important role. Abnormalities occurred in the functions related with the initiation of innate immunity and adaptive immunity in the early phase, and therefore, the system network performance has a brief increase. However, with the disease progression, the immune system is insufficient to compensate for abnormal function. Therefore, the functional level of the immune system is an important factor in determining the risk of lung adenocarcinoma and an important indicator of the early diagnosis of lung adenocarcinoma.

### Functional pathway imbalance score

We use Eq. 3 to calculate the variance score for each enriched functional pathway. To investigate whether the abnormality of these functional levels is significantly present in different phases, we analyse the imbalance scores in each of the three groups of samples. Finally, we select 12 significant pathways in the ANOVA analysis, as shown in Table 2.

**Table 2:** The functional significance in ANOVA

Pathway term	P value
Primary.immunodeficiency	1.08E−05
B.cell.receptor.signalling.pathway	5.37E−05
Haematopoietic.cell.lineage	0.000310509
T.cell.receptor.signalling.pathway	0.001363854
Fc.epsilon.RI.signalling.pathway	0.002015294
Regulation.of.actin.cytoskeleton	0.004150605
Non.small.cell.lung.cancer	0.006087547
Leukocyte.transendothelial.migration	0.01039866
Cell.adhesion.molecules	0.01324628
Natural.killer.cell.mediated.cytotoxicity	0.0139198
Fc.gamma.R.mediated.phagocytosis	0.01575172
NF.kappa.B.signalling.pathway	0.01622258

The first column is the functional term, and the second column contains the significant P values in the ANOVA analysis

**Fig. 4:**
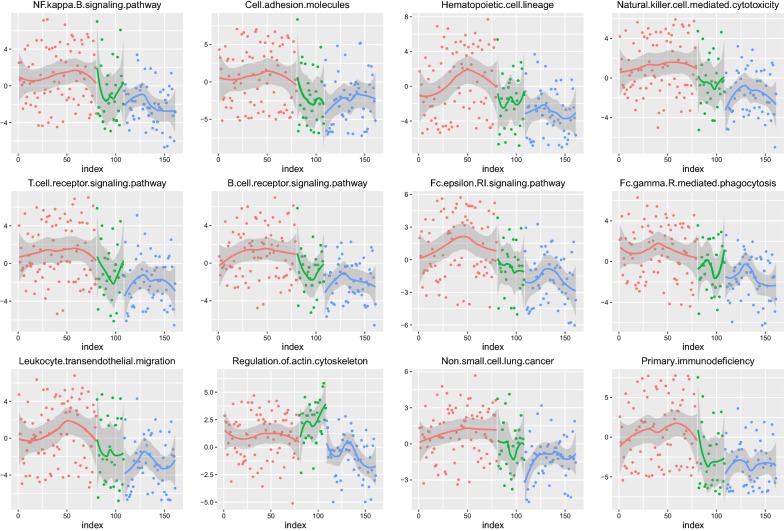
The pathway dynamic change across the three phases. We use red, green, and blue to mark normal, early phase and late phase, one by one. According to the dynamic change in the pathway in each phase, we use non-parametric linear fitting to observe the trend

We can see that these 12 pathways have obvious differences in the three phases; the P value is less than 0.05. To more intuitively analyse the imbalance of each pathway in these three phases’ samples, we use the scatter plot to visualize the dynamic process of 12 pathways, as shown in Fig. 4.

**Fig. 5:**
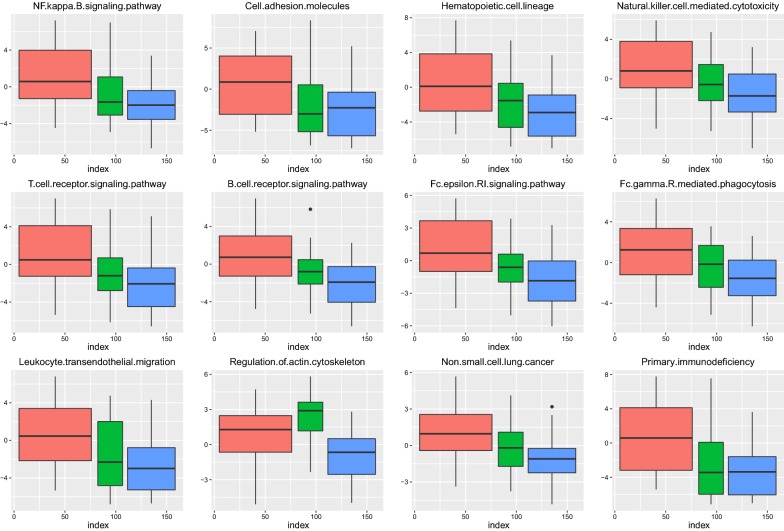
The boxplot visualization. The 12 pathways in three different phases of the sample in the scoring boxplot graph; the graph demonstrates the median and confidence interval. The three phase groups are also marked with red, green, and blue

It can be seen that the functional levels in the normal phase are inconspicuous and fluctuate near 0; the functional levels from the early phase begin to produce a significant fluctuation. To further clarify the direction of imbalance and variation of each pathway in the three phases, we compare the average distribution of each pathway through the boxplot visualization, as shown in Fig. 5.

It can be intuitively seen that in most of the pathways, the scores of the three groups are gradually reduced. This suggests that these levels of function are inhibited during the progression of lung cancer, and the level of immune system function is reduced by cancer invasion. The functional level of the actin cytoskeleton pathway presents an upward trend in the early phase, and then, with the progression of cancer, the functional level further declines. This may be caused by the stress response of the body under the stimulation of the cancer. Therefore, functional abnormalities can be found to occur in the early phase of lung cancer, and thus, the use of genes regulating these functions as a diagnostic feature can achieve the early diagnosis of lung cancer.

### Early-phase diagnostic marker screening using the RFE algorithm

We found that the functional level of variation occurs in the pathways enriched from early- and late-phase specific genes, compared with the normal state. To achieve the early diagnosis of lung cancer and identify the genetic markers that significantly change with the progression of lung cancer, we used the recursive feature elimination algorithm on the 198 genes. Finally, 12 genes were screened as diagnostic markers.

**Fig. 6:**
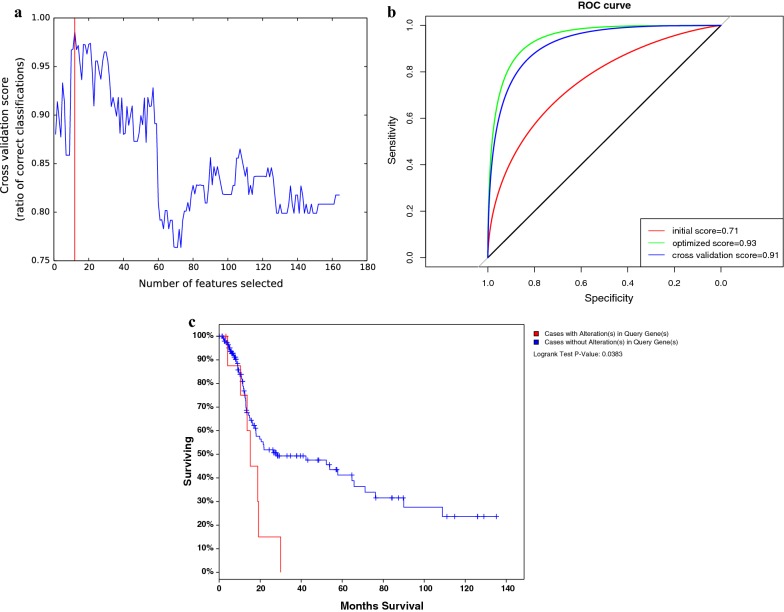
The performance of biomarkers. **a** The horizontal axis is the number of selected features. The vertical axis is the corresponding accuracy. **b** The red curve is the initial model accuracy without optimization. The green curve is the accuracy of the model after feature selection and parameter optimization. The blue curve is the average accuracy calculated using a fivefold cross-validation method. **c** The survival analysis. The horizontal axis is the survival time (month). The vertical axis is the percentage of patients in different groups

Figure 6a shows the RFE algorithm optimization process; when the number of feature genes is 12, the model has the highest precision. Therefore, we use these 12 genes as the features to train the diagnostic model.

### Risk diagnostic model construction based on early screening genes

We take 12 genes as features, using an SVM (support vector machine) to construct the classification model. During the initialization process, we set all the model parameters as the default parameters and intensively test the initial accuracy of the model in the training set. The parameter optimization process utilizes the grid search algorithm and iterates to find the optimal combination of parameters. The final model classification prediction results are shown in Fig. 6b.

Figure 6b illustrates the classification efficiency of the model by ROC curve evaluation. In the process of cross-validation, we randomize samples each time, taking four to do the training. For a prediction graph, the horizontal axis is the false positive rate; the vertical axis is a true positive rate, and the final model achieves an average accuracy of 0.91. The five-time cross validation accuracy is similar to the accuracy of the model in the training set, indicating that the model does not exhibit significant over-fitting. Using our predictive model of training, we can achieve early prediction of lung adenocarcinoma and distinguish benign and malignant progression. Notably, it not only provides new insight into the pathogenesis of lung adenocarcinoma but also suggests a new diagnostic marker or therapeutic target.

### Survival analysis for validation

We downloaded samples of lung adenocarcinoma from the TCGA database as independent data for survival analysis. We identified 12 marker genes that could predict the diagnosis of early lung cancer patients, suggesting that these genes were significantly altered by disease signal stimulation in the early stage of cancer. After the functional analysis, we found that these genes were involved in the inherent immune and adaptive immune system; therefore, we speculate that with the progression of lung cancer, the patient’s immune system followed with abnormal functional levels. To investigate whether these genes affected the survival of the patients by interfering with the immune level of the patients, we set the samples with variations of these 12 genes to be considered as high-risk groups, and the samples with no significant variations were set as the low-risk group and combined with Cox regression. The survival analysis results are shown in Fig. 6c. The significance (P) value was 0.03, indicating that the two groups of samples present significant differences in the survival level.

## Discussion

X-ray is so far the primary tool for the clinical diagnosis of lung cancer, but for early lung cancers, misdiagnosis is unavoidable because of its low sensitivity. However, another method to confirm the diagnosis is tissue puncture, which is also a diagnostic tool that may cause cancer cells to spread and metastasize. Lung adenocarcinoma in the early stage is under restriction in local lesions by using ultrasound or X-ray, and other detective methods may lead to a missed diagnosis [25]. However, in the early stages of cancer development, the level of molecular expression has changed. This change occurs because the microenvironment of cancer tissue lesions is usually accompanied by the local inflammatory response, which activates the body’s stress response and immune response. In the initial stage, under the regulation of innate immunity and adaptive immunity, multiple genes are differentially expressed to compensate for function and resist cancer [26]. However, the low sensitivity and specificity impede the broad application of these biomarkers in clinics. Therefore, to develop new methods and novel diagnostic biomarkers is necessary for the early detection of lung adenocarcinoma.

In the late phase of lung cancer, malignancy is increased due to the impact of decompensation. Therefore, more genes tend to present diverse expression. At the same time, due to the different stages of pathogenesis and degrees of malignancy, the gene expression patterns are remarkably different [27]. Hence, precisely distinguishing early- and late-phase patients from gene expression patterns as well as the level of functional abnormalities is a milestone for the early diagnosis of lung adenocarcinoma.

**Fig. 7:**
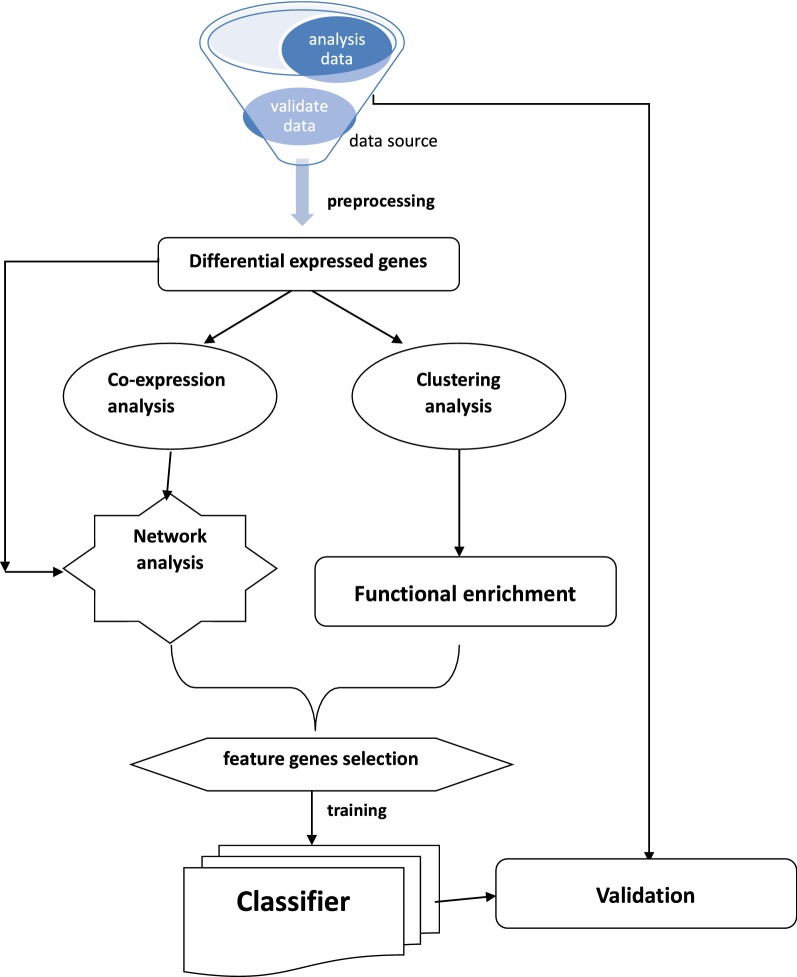
Workflow summary for analysis process. The flow chart from the datasets downloaded, different stages, methods used for different analyses, to gene sets generated from one analysis and used for another analysis

In this study, we identified early- and late-phase specific genes and the shared genes that are always differentially expressed across the cancer process. The flow chart of the whole study was shown in Fig. 7. Combined with unsupervised clustering analysis, we found that there were significant differences in the expression patterns of 164 shared genes between early-phase lung cancer (stage I) and advanced lung cancer (stage II–IV). To further discover the specificity between the two phases, we separately analysed each phase-specific gene set. We used the Pearson correlation coefficient to calculate the similarity between any two genes in each phase-specific gene set [13]. By using the relevant gene relationship, we constructed the co-expression network and analysed the topology of the network. By comparing the specific characteristics of the co-expression networks, we found that the network structure changes when the degree of malignancy increases [28].

With the rise in the degree of malignancy of lung cancer, the average shortest path of the system network gradually increases, suggesting that the network is becoming looser, and the signal transmission efficiency is gradually reducing [29]. Similarly, the closeness centrality reduces as the progression of the disease also prompts the network performance to decline [30]. Changes in the network structure suggest that in the process of lung cancer progression, the interactions between the genes exhibit dynamic changes under the regulation of the immune system. This interaction under the immune response reflects the complex changes in the biological system from stress compensation to decompensation during tumour progression [31]. Some genes embody the correlation at an early stage, but in the process of tumour mutation, the inherent correlation between genes is absent, suggesting that at least one of them is a tumour-related gene and is abnormal in expression level due to mutation [32]. Beyond that, some genes are not related to the early phase, but in the late phase, they reflect the correlation, suggesting that these genes are likely to have a functional consistency, and they are initially in a silent state. Only when the activated gene is abnormal does the silent gene assume its role, which results in a new correlation.

We analysed the functional enrichment of each phase-specific gene, and the results showed that the mechanism of immune regulation in the process of lung adenocarcinoma dramatically changes. Abnormal immune systems include innate immunity, T/B lymphocyte-regulated specific adaptive immunity, natural killer-regulated nonspecific immunity, and other infections and inflammation-related functions. This further suggests that the abnormalities of the immune system are essential causes of progression of lung adenocarcinoma [33]. Compared with the normal phase, early phase-related genes and late phase-related genes are differentially expressed. To further screen out the markers with diagnostic efficacy from these genes, we screened them through machine learning algorithms. Finally, we screened 12 genes with diagnostic efficacy and used these genes as features to construct a diagnostic model. The model’s diagnostic accuracy for early lung cancer patients reaches 91%.

The innovation of this paper is about identifying phase-specific genes and functions of lung adenocarcinoma, which serves as a guide for selecting personalized diagnostic markers or therapeutic targets. The limitation of this paper is that the experimenters are not able to further discover the co-expression of gene dynamics. Actually, more than two genes can be involved in the same function, which means using “gene cluster” instead of “gene pair” might provide more information. However, to guarantee the correlation with a gene cluster is not randomness, we have to rely on adequate annotation data to prove genes correlation. Meanwhile, using the functionality as the feature, the explanation of the specific mechanism driving changes to different stages can become more intuitive. If it is possible to screen out the stabilized gene pairs, the method is still more stable than the detection methods using single genes as features.

## Conclusions

In this study, we demonstrated that the co-expression and its reconstruction between genes reflect the progress of genes associated with the progression of lung adenocarcinoma and as the feature to distinguish lung cancer with different levels of risk. Based on co-expression similarity and construction of a diagnostic model, we identify an early diagnostic biomarker of lung adenocarcinoma. Overall, this may provide a new insight into the diagnosis and prediction of lung cancer.

## Abbreviations

NSCLC: non-small cell lung cancer
TCGA: the cancer genome atlas
RFE: recursive feature elimination
SVM: support vector machine
ROC: receiver operating characteristic curve
